# Effects of instrument-assisted soft tissue mobilization combined with exercise on knee joint muscle activity during walking in patients with moderate knee osteoarthritis: A randomized controlled clinical trial

**DOI:** 10.1038/s41598-025-28418-3

**Published:** 2025-12-29

**Authors:** Bahare Jafarsalehi, Sahar Boozari, Giti Torkaman

**Affiliations:** Department of Physiotherapy, Faculty of Medical Sciences, Tarbiat Modares University, Gisha, Jalal St., Tehran, 1411713116 Iran

**Keywords:** Osteoarthritis, Myofascial release therapy, Gait, Electromyography, Muscle strength, Health care, Medical research, Rheumatology

## Abstract

Knee osteoarthritis (KOA) is a degenerative joint disorder characterized by pain and stiffness, often leading to compensatory increases in muscle activity. Instrument-assisted soft tissue mobilization (IASTM) is an emerging manual therapy with potential benefits in soft tissue release and pain management. This study investigated the effects of IASTM combined with exercise therapy on muscle activity and clinical outcomes in individuals with KOA. Thirty KOA patients were randomly allocated to either an IASTM plus exercise group or a sham IASTM plus exercise group. The intervention consisted of four sessions over two weeks. Electromyographic (EMG) activity was assessed pre- and post-treatment using mean root mean square (RMS) amplitude values and co-contraction indices of selected lower limb muscles during preferred and fast walking. Pain intensity, knee and ankle strength, and self-reported disability were also evaluated. Significant group*time interactions were observed for the vastus medialis, semitendinosus, and tibialis anterior, with post-treatment EMG activity decreasing in the IASTM group and increasing in the sham group. All mean RMS values and co-contraction indices were significantly higher during fast walking. A significant speed*time interaction for the vastus lateralis revealed more pronounced post-treatment reductions during fast walking, with a trend toward decreased activity in the IASTM group and increased activity in the sham group. Both groups demonstrated significant improvements in pain, disability, and muscle strength; however, the IASTM group showed greater improvements, supported by significant interaction effects. Correlation analysis revealed that EMG activity in the rectus femoris, vastus medialis, and biceps femoris was moderately and positively associated with pain levels. The rectus femoris and medial gastrocnemius also showed inverse associations with muscle strength. Although both groups showed improvements in pain, strength, and disability, greater gains were observed in the IASTM group. Notably, IASTM combined with exercise therapy also reduced EMG activity. This reduction, alongside increased strength, suggests enhanced neuromuscular efficiency, which may decrease intra-articular loading associated with elevated muscle activation—commonly observed in KOA patients—while supporting joint stability. These findings highlight the potential benefit of incorporating soft tissue mobilization techniques alongside exercise therapy in KOA rehabilitation programs. **Trial registration **This trial was registered on 14 January 2022 at https://www.irct.behdasht.gov.ir with the registration code IRCT20201128049511N3. It is also accessible via the WHO trial search portal at: https://trialsearch.who.int/Trial2.aspx?TrialID=IRCT20201128049511N3.

## Introduction

Knee osteoarthritis (KOA) is among the most common degenerative joint disorders worldwide, affecting millions of individuals and placing a considerable burden on healthcare systems. Clinically, KOA is characterized by joint pain, crepitus, stiffness, and swelling, leading to functional impairments, disability, and reduced quality of life^1–3^. Although commonly associated with cartilage degeneration, KOA also involves periarticular soft tissues, including surrounding muscles. Patients frequently report soft tissue discomfort, reduced flexibility, and muscle weakness, all of which contribute to functional limitations^4,5^. These observations underscore the importance of addressing muscle function as part of KOA management.

Muscle weakness in KOA may result from pain-induced neuromuscular inhibition and impaired muscular performance, ultimately compromising joint stability^6–8^. As a compensatory response, individuals with KOA commonly exhibit elevated muscle activation and co-contraction to maintain joint support, as demonstrated in kinesiological electromyography (EMG) studies^9–14^. While this strategy may offer short-term stabilization, sustained overactivity can increase intra-articular loading, intensify pain, accelerate cartilage degeneration, and contribute to structural deterioration^9–17^. Consequently, interventions that reduce excessive muscle activation and abnormal co-contraction—while concurrently enhancing muscle strength to support joint stability—are essential for mitigating symptoms and slowing disease progression.

Conservative treatment strategies for KOA, including electrotherapy, exercise therapy, and manual therapy, primarily aim to relieve symptoms, improve function, and delay surgical intervention^18,19^. Among these, instrument-assisted soft tissue mobilization (IASTM) is an emerging manual technique that uses specialized tools to deliver mechanical force to soft tissues, reducing physical effort for clinicians while potentially enhancing therapeutic outcomes. Reported effects include improved tissue mobility, pain relief, and myofascial release, potentially mediated by increased blood flow, reduced fascial adhesions, and decreased tissue viscosity^20–23^. Notably, IASTM is rarely used as a standalone intervention; it is typically integrated into multimodal treatment plans alongside exercise therapy or electrotherapy to optimize clinical benefit^20,22,24–27^. In the context of KOA, previous studies have demonstrated favorable effects of combined IASTM protocols with exercise or electrotherapy on pain reduction, joint range of motion, and overall health status^24,26,27^. Nonetheless, these investigations have primarily focused on clinical outcomes, with limited exploration of neuromuscular responses that may reflect underlying treatment-related adaptations.

Given the significant role of muscle activity in functional performance and symptom management among individuals with KOA, this study aimed to evaluate the effects of IASTM combined with exercise therapy on mean root mean square (RMS) amplitude values and co-contraction indices of selected lower limb muscles during gait—a fundamental component of daily mobility. These variables were selected due to their relevance in KOA, where elevated activation and co-contraction often emerge as compensatory responses to pain and joint instability—patterns that may contribute to inefficient movement and joint deterioration. To capture treatment effects under varying demands, assessments were conducted at both preferred and fast walking speeds, since fast walking imposes greater neuromuscular stress^28^ and may reveal subtle changes in muscle activation not detectable at lower speeds following treatment. In addition to EMG outcomes, pain intensity, lower limb strength, and self-reported functional disability—assessed using the Lequesne Algofunctional Index (LAI)—were evaluated to explore their associations with muscle activation changes, offering complementary insight into the clinical relevance of the EMG findings.

We hypothesized that IASTM combined with exercise would lead to greater reductions in muscle activation, co-contraction, and pain, alongside improvements in strength and functional outcomes, compared to exercise alone. This hypothesis is grounded in proposed mechanisms by which IASTM may disrupt myofascial adhesions, enhance local circulation, and modulate pain—thereby facilitating neuromuscular efficiency and amplifying the therapeutic effects of exercise. From a clinical perspective, incorporating EMG alongside conventional symptom-based assessments may strengthen the rationale for integrating soft tissue mobilization techniques into multimodal rehabilitation programs for KOA, by highlighting their potential to improve both clinical outcomes and underlying muscle activation levels.

## Methods and materials

### Study design

This study was a parallel, randomized, controlled, double-blind clinical trial. A repeated-measures design was employed to evaluate the effects of two treatment conditions—IASTM combined with exercise therapy versus sham treatment combined with exercise—across two walking speeds (preferred and fast) and at two time points (pre-treatment and post-treatment). Lower limb EMG activity during gait served as the primary outcome, while pain intensity, muscle strength, and disability were assessed as secondary outcomes.

### Participants

Thirty-three individuals diagnosed with unilateral KOA were recruited voluntarily. Inclusion criteria were: age > 40 years; diagnosis of unilateral moderate KOA (Kellgren–Lawrence grades 2 or 3); ability to walk independently without assistive devices; peak pain intensity between 3 and 7 on the visual analog scale (VAS) within the previous 24 hours; positive Clarke’s test; and body mass index (BMI) between 18.5 and 29.9. Exclusion criteria included: any permanent orthopedic, neurological, or rheumatologic condition affecting the lower limbs or lower spine; intra-articular knee injections within the past six months; severe knee deformity; candidacy for total knee arthroplasty; or a leg length discrepancy greater than 1.5 cm^29,30^.

Sample size estimation was based on pilot data using a partial eta-squared value of 0.072 for the mean RMS amplitude of the rectus femoris (RF), with an alpha level of 0.05 and beta level of 0.2. Calculations were performed using G*Power software^31^, resulting in a required sample of 28 participants. To account for potential dropout, recruitment was increased by 15%, yielding a total of 33 participants. Three individuals withdrew for personal reasons prior to randomization; the remaining 30 participants were randomly assigned to either the IASTM or sham group.

Randomization was conducted using a stratified block design with a 1:1 allocation ratio, based on two stratification factors: pain intensity (VAS 3–5 vs. 6–7) and knee osteoarthritis severity (Kellgren–Lawrence grade 2 vs. 3). To ensure allocation concealment, an independent researcher not involved in data collection performed the randomization. Double blinding was maintained throughout the study: the outcome assessor remained unaware of group assignments, and participants were also blinded to their allocated group. The participants were informed that the study involved a physiotherapy approach using specialized tools, without disclosure of whether they were receiving the actual or sham intervention. To minimize the risk of participants identifying their allocation, each group received the intervention on separate days.

The study was approved by the Ethics Committee of Tarbiat Modares University (Approval ID: IR.MODARES.REC.1400.201; https://ethics.research.ac.ir) and was prospectively registered on January 14, 2022, in the Iranian Registry of Clinical Trials (IRCT20201128049511N3; https://irct.behdasht.gov.ir), a WHO-recognized primary registry. All procedures were conducted in accordance with relevant guidelines and regulations, including the Declaration of Helsinki. All participants provided written informed consent before enrollment.

**Fig. 1:**
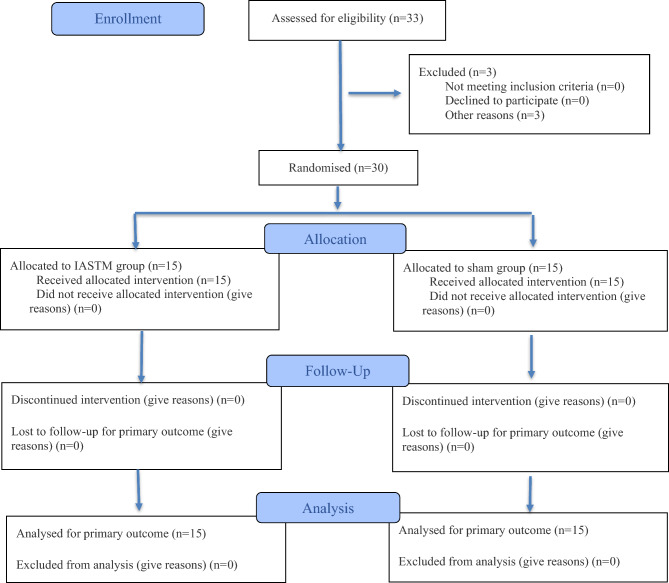
CONSORT 2010 flow diagram of the study.

The study adhered to the CONSORT guidelines for reporting randomized trials^32^. The participant flow diagram is presented in Fig. 1.

### Test procedure

Assessment and treatment sessions were conducted between May 2022 and August 2023 at the Research and Treatment Center for Movement Disorders, Tarbiat Modares University. Baseline evaluations prior to the intervention included measurements of pain intensity, self-reported functional disability, and lower limb muscle strength. In addition, surface EMG recordings were obtained during gait at both preferred and fast walking speeds to capture neuromuscular responses under varying functional demands.

#### Clinical symptom evaluation

Pain intensity during walking over the past 24 hours was assessed using a 100-mm VAS, and self-reported functional disability was measured using the LAI. The LAI consists of 24 items, with a maximum score of 14 indicating severe functional impairment^33^. In this study, a Persian version of the LAI, previously demonstrated to be reliable and valid, was utilized^34^.

#### Muscle strength assessment

Maximum voluntary isometric contraction (MVIC) of lower limb muscles was assessed using a handheld dynamometer (Lafayette Instrument Co., Lafayette, IN, USA). Each isometric contraction was maintained for three seconds and repeated three times, with a 60-second rest interval between trials. Participants completed a standardized warm-up and familiarization process prior to testing, and verbal encouragement was provided to ensure maximal effort. Knee extensor strength was measured in a seated position with the knee flexed at 90°, and the dynamometer positioned just above the ankle. Knee flexor strength was assessed in the prone position, with the device placed above the ankle on the posterior aspect of the lower leg. Plantar flexor strength was evaluated in the prone position with the device positioned against the sole of the foot, while dorsiflexor strength was measured in the supine position with the dynamometer placed on the dorsum of the foot^35^. For each trial, the mean force generated during the three-second contraction was recorded and normalized to body mass. The average force across three trials for each muscle group was then calculated for subsequent analysis.

#### Surface EMG acquisition during walking and signal processing

Surface EMG data were collected using a wireless 16-channel system (Aktos, Mayon Inc., Switzerland) and Nexus software (Vicon Nexus, Oxford, UK), with a sampling frequency of 1200 Hz and bandwidth of 10–500 Hz. To ensure proper electrode adhesion and signal quality, the skin was shaved and cleansed with rubbing alcohol before electrode placement. Disposable surface electrodes (Shanghai INTCO Electrode Manufacturing Co., Ltd., China) were placed on the affected limb in accordance with SENIAM guidelines^36^, targeting the rectus femoris (RF), vastus medialis (VM), vastus lateralis (VL), semitendinosus (ST), biceps femoris (BF), tibialis anterior (TA), medial gastrocnemius (MG), and lateral gastrocnemius (LG). Placement accuracy was verified through visual inspection of EMG signals during MVIC testing and adjusted as necessary.

Participants completed three walking trials on a 10-meter walkway at both preferred and fast speeds, each repeated three times. Preferred speed was defined as the self-selected comfortable pace, while fast speed required walking as quickly as possible without running. EMG data were extracted from the stance phase of the gait cycle, which represents the load-bearing period and typically imposes greater stress on the joints and neuromuscular system in individuals with KOA. To identify this phase, retro-reflective markers were positioned according to the Vicon Plug-in-Gait lower body model, and motion data were recorded using a Vicon system (Vicon, Oxford, UK) equipped with eight Vero cameras (2.2 MP) at a sampling frequency of 120 Hz. The stance phase—defined as the interval from heel contact to toe-off—was determined based on marker trajectories captured during the middle step of the walkway^37^. EMG and motion capture data were then synchronized to enable precise identification of muscle activity during the stance phase.

EMG signals were processed using a custom MATLAB script (MathWorks Inc., Natick, MA, USA). Signals were full-wave rectified, low-pass filtered using a fourth-order Butterworth filter at 5 Hz, and normalized to EMG recordings obtained during MVIC testing. RMS values were calculated using a 20-ms moving window, and mean RMS amplitudes were derived from the stance phase. Co-contraction indices were calculated for four muscle pairs (VM–ST, VL–BF, TA–MG, and TA–LG) using the following formula^38^:

$$Co-contraction index=\frac{1}{100}\sum\limits_{i=0}^{100}\left[\frac{{Lower EMG}_{i}}{{Higher EMG}_{i}} \times ({Lower EMG}_{i}+{Higher EMG}_{i})\right]$$

All EMG variables were averaged across three stance phases, each extracted from the middle step of the walkway, for both walking speeds.

### Intervention

**Fig. 2:**
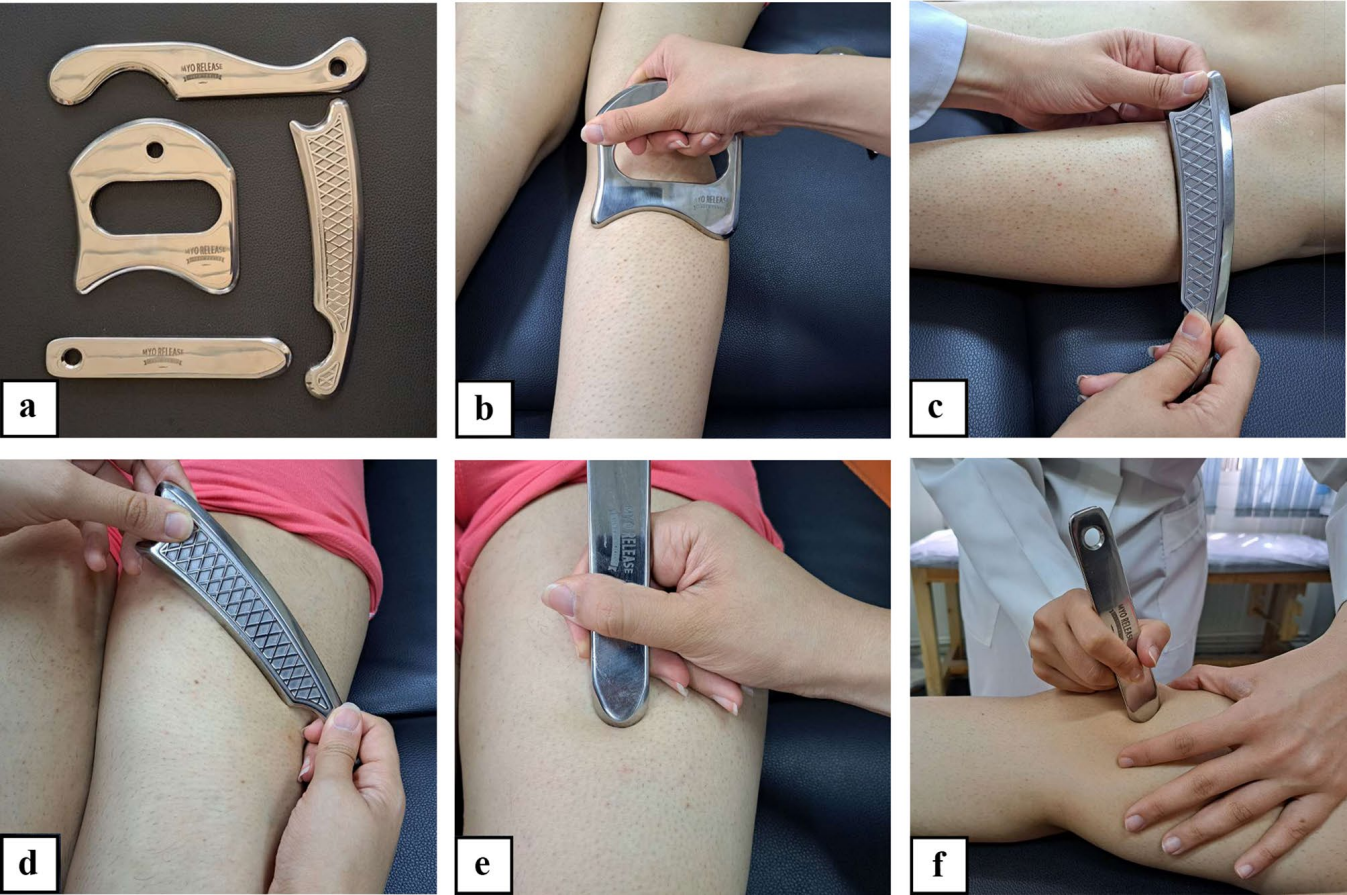
IASTM tools (**a**) and techniques: sweeping (**b, c**), fanning (**d**), brushing (**e**), and framing (**f**).

One day following the initial assessment, participants returned for the intervention, which was administered by licensed physiotherapists with a minimum of two years of experience in IASTM. Given that all participants had unilateral KOA, the treatment was specifically applied to the affected limb. The treatment protocol adhered to the HawkGrips methodology and incorporated four techniques: sweeping, fanning, brushing, and framing^22,39^. Two sets of instruments were employed—HawkGrips (HGPro Multi-Tool, USA) and Myorelease tools (IS-3, IS-4, IS-22, Iran) (Fig. 2).

Each session commenced with sweeping (long, parallel strokes using the broad edge of the instrument) and fanning (arc-shaped movements pivoting around a fixed point) to evaluate soft tissue irregularities. Periarticular structures and major lower limb muscles—including the quadriceps, hamstrings, gastrocnemius, and tibialis anterior—were palpated and assessed. Regions exhibiting sub-surface irregularities or vibration sensations upon instrument movement, as well as areas associated with patient-reported discomfort, were selected as target sites for intervention. Treatment strokes were applied in the following sequence: sweeping and fanning, followed by brushing (fine, parallel strokes using the narrower edge of the instrument) and framing (short strokes around the patellar margin)^22,39^. Each technique was applied for 60–90 seconds per symptomatic area, maintaining a bevel angle between 30° and 60°. An emollient was used throughout to facilitate smooth instrument gliding. Total treatment time per session was approximately 15 minutes.

The IASTM intervention was delivered across four sessions over a two-week period (two sessions per week), based on prior evidence supporting its immediate and short-term effects on pain reduction, joint mobility, and muscle performance^26,40,41^.

For the sham intervention, the same instruments were used but applied with minimal pressure, gently contacting the skin without delivering any therapeutic strokes. Contact duration matched that of the intervention group to maintain procedural consistency.

Immediately following each IASTM or sham session, participants completed a standardized exercise program comprising stretching and strengthening exercises for KOA patients. This integration reflects typical clinical practice, wherein IASTM is administered adjunctively rather than in isolation^25^. The program comprised stretching and strengthening exercises. Stretching targeted the quadriceps, hamstrings, and gastrocnemius muscles, with each stretch held for 30 seconds and repeated four times. Strengthening exercises included quadriceps setting, active straight leg raises, seated knee extensions, and supine isometric contractions for the hamstrings, gastrocnemius, and tibialis anterior, performed with therapist assistance when needed. Strengthening was performed in sets of 10 repetitions, with each held for a maximum of 10 seconds^42,43^.

A follow-up assessment of pain intensity, functional disability, muscle strength, and EMG parameters was conducted 48 hours after completion of the final treatment session.

### Statistical analysis

Data distribution was evaluated using the Shapiro–Wilk test. All variables followed a normal distribution, with the exception of VAS scores. Accordingly, baseline group comparisons were performed using independent-samples t-tests for normally distributed variables and the Mann–Whitney U test for VAS.

Despite VAS non-normality, repeated-measures ANOVA was employed for all outcomes due to its robustness to moderate violations of normality. Other key assumptions—including homogeneity of variances and equality of covariances—were satisfied, as confirmed by Levene’s test and Box’s M test, respectively. For EMG parameters, a mixed-design ANOVA was conducted with group (IASTM vs. sham) as the between-subject factor, and walking speed (preferred vs. fast) and time (pre- vs. post-treatment) as within-subject factors. For clinical outcomes, a separate mixed-design ANOVA was applied to the VAS, LAI, and strength measures, incorporating one between-subject factor (group: IASTM vs. sham) and one within-subject factor (time: pre- vs. post-treatment), as these variables were assessed only before and after the intervention and not across different walking speeds. Effect sizes were reported using partial eta squared (η^2^p), with thresholds of 0.01, 0.06, and 0.138 interpreted as small, moderate, and large effects, respectively^44^.

Pearson’s two-tailed correlation analysis was used to examine associations between EMG variables and clinical measures (VAS, LAI, and muscle strength). The correlation of each muscle’s EMG activity with the strength of its anatomically corresponding muscle group was also assessed. Additional correlations were calculated between VAS and LAI, VAS and strength, and LAI and strength. Co-contraction indices were excluded from correlation analysis with strength, as they reflect paired muscle activity and cannot be directly attributed to the performance of a single muscle group. Correlation coefficients (r) were classified as small (0.10–0.29), moderate (0.30–0.49), or large (0.50–1.00)^44^.

All the statistical analyses were performed using SPSS software, with a significance level set at p < 0.05.

## Results

All 30 participants successfully completed the treatment protocol and were included in the final analysis. Baseline demographic characteristics are presented in Table 1, with no statistically significant differences observed between the two groups (p > 0.05).

**Table 1:** Demographic characteristics of the participants (Mean ± SD).

**Variable**	**IASTM (n = 15)**	**Sham (n = 15)**	**p-value**
Sex	13 Female, 2 Male	12 Female, 3 Male	N/A
Age (years)	57.73 ± 8.54	58.27 ± 7.36	0.277
Height (m)	1.62 ± 0.07	1.59 ± 0.07	0.591
Weight (kg)	72.42 ± 10.93	70.16 ± 11.75	0.904
BMI	27.46 ± 2.95	27.59 ± 2.82	0.856

Moreover, no significant baseline differences were observed for EMG, strength, or LAI outcomes (independent-samples t-tests), nor for VAS scores (Mann–Whitney U test), confirming comparability between groups prior to intervention.

### Pain, disability, and muscle strength outcomes

Results of the mixed ANOVA for pain (VAS), disability (LAI), and muscle strength are summarized in Table 2.

**Table 2:** Descriptive statistics and ANOVA results for pain, LAI, and muscle strength before and after treatment.

Variables	Before treatment		After treatment		Group effect	Time effect	Interaction effect
	IASTM	Sham	IASTM	Sham			
VAS (cm)	4.60 (0.91)	4.46 (0.63)	1.66 (1.67)	3.13 (0.99)	0.067 (1.115)	˂0.001^*^ (0.781)	0.001^*^ (0.334)
LAI	9.00 (2.54)	7.66 (3.55)	5.86 (1.89)	6.10 (3.21)	0.600 (0.010)	˂0.001^*^ (0.873)	˂0.001^*^ (0.432)
Knee extensors	0.078 (0.01)	0.074 (0.01)	0.099 (0.02)	0.081 (0.01)	0.065 (0.116)	˂0.001^*^ (0.416)	0.038^*^ (0.145)
Knee flexors	0.078 (0.01)	0.077 (0.01)	0.099 (0.01)	0.083 (0.02)	0.122 (0.083)	˂0.001^*^ (0.357)	0.029^*^ (0.160)
Plantar flexors	0.084 (0.01)	0.085 (0.02)	0.105 (0.01)	0.091 (0.02)	0.244 (0.048)	˂0.001^*^ (0.398)	0.020^*^ (0.179)
Dorsi flexors	0.092 (0.01)	0.086 (0.01)	0.096 (0.03)	0.089 (0.01)	0.342 (0.032)	0.422 (0.023)	0.920 (0.001)

Footnote: Data are presented as the mean (SD), with p-values and partial eta squared (η^2^p) values.

VAS: visual analog scale; LAI: Lequesne algofunctional index.

Strength values normalized to body mass; unit not applicable.

^*^Indicates p < 0.05.

Significant main effects of time and group*time interactions were observed for VAS, LAI, knee extensor strength, knee flexor strength, and plantar flexor strength. As presented in Table 2, both groups showed improvements over time; however, the significant interaction effects suggest that the magnitude of improvement differed between the IASTM and sham groups, with the IASTM group showing more pronounced improvements across these outcomes. VAS scores declined by 63.91% in the IASTM group compared to 29.82% in the sham group. LAI scores improved by 34.89% and 20.37% in the IASTM and sham groups, respectively. Strength gains were also greater in the IASTM group: knee extensor and flexor strength increased by 26.92%, while the sham group showed increases of 9.46% and 7.79%, respectively. Plantar flexor strength rose by 25.00% in the IASTM group versus 7.06% in the sham group. In contrast, dorsiflexor strength exhibited no significant interaction effect, with comparable improvements recorded in both groups (4.35% for IASTM, 3.49% for sham).

### Surface EMG outcomes

Descriptive statistics and mixed ANOVA results for mean RMS values and co-contraction indices are presented in Table 3.

**Table 3:** Descriptive statistics and ANOVA results for mean RMS values and co-contraction indices at preferred and fast walking speeds (pre- and post-treatment).

Variables	Speed	Before treatment		After treatment		Group effect	Time effect		Speed effect	
		IASTM	Sham	IASTM	Sham					
VM	P	52.12 (21.71)	53.46 (27.63)	31.84 (17.41)	71.90 (49.16)	0.046^*^ (0.139)	0.396 (0.027)		˂0.001^*^ (0.555)	
	F	72.84 (38.00)	71.91 (32.30)	38.86 (26.76)	86.15 (49.30)					
VL	P	45.13 (21.78)	55.81 (22.74)	34.84 (16.33)	60.70 (41.89)	0.070 (0.125)	0.437 (0.024)		˂0.001^*^ (0.598)	
	F	64.89 (34.92)	67.51 (24.44)	46.04 (22.28)	68.17 (30.28)					
RF	P	61.87 (27.21)	60.73 (25.93)	63.69 (11.66)	68.24 (48.95)	0.931 (0.001)	0.858 (0.001)		˂0.001^*^ (0.632)	
	F	78.20 (27.50)	75.13 (25.61)	82.35 (14.05)	75.41 (49.50)					
ST	P	26.03 (15.75)	24.29 (15.50)	21.82 (9.65)	36.79 (19.51)	0.220 (0.055)	0.366 (0.030)		˂0.001^*^ (0.475)	
	F	30.98 (18.22)	28.16 (15.78)	25.63 (10.05)	38.22 (18.80)					
BF	P	28.02 (13.07)	29.30 (16.61)	23.09 (11.34)	34.45 (17.48)	0.221 (0.055)	0.929 (0.001)		˂0.001^*^ (0.637)	
	F	33.52 (14.69)	35.82 (18.38)	29.21 (13.16)	38.79 (20.68)					
MG	P	45.55 (21.43)	63.11 (26.25)	42.25 (14.46)	52.70 (18.96)	0.062 (0.138)	0.144 (0.077)		˂0.00^*^ (0.579)	
	F	62.95 (33.75)	75.98 (30.07)	52.79 (19.67)	63.38 (23.19)					
LG	P	44.67 (21.01)	49.91 (29.26)	47.70 (24.13)	61.18 (37.00)	0.327 (0.039)	0.395 (0.029)		˂0.001^*^ (0.668)	
	F	57.30 (31.44)	61.63 (31.50)	55.57 (25.94)	71.06 (39.37)					
TA	P	42.32 (13.63)	48.19 (19.09)	34.09 (11.22)	53.83 (25.62)	0.071 (0.125)	0.323 (0.039)		˂0.001^*^ (0.828)	
	F	59.51 (16.70)	63.81 (23.86)	46.05 (16.74)	66.31 (27.25)					
VM-ST	P	33.53 (25.06)	33.85 (25.40)	26.80 (17.96)	48.77 (31.32)	0.176 (0.067)	0.731 (0.004)		˂0.001^*^ (0.608)	
	F	43.08 (33.28)	42.02 (28.02)	31.95 (20.27)	53.58 (31.10)					
VL-BF	P	35.24 (24.51)	48.75 (32.93)	31.20 (16.01)	64.70 (50.47)	0.065 (0.154)	0.585 (0.011)		˂0.001^*^ (0.621)	
	F	45.49 (29.51)	57.92 (34.46)	39.48 (16.98)	70.78 (48.68)					
TA-MG	P	58.08 (27.72)	61.99 (32.84)	43.63 (18.88)	69.37 (43.14)	0.148 (0.082)	0.397 (0.029)		˂0.001^*^ (0.717)	
	F	77.92 (35.75)	81.63 (41.84)	56.22 (24.51)	82.08 (41.83)					
TA-LG	P	54.02 (27.57)	58.78 (29.04)	42.67 (19.97)	73.22 (47.76)	0.142 (0.084)	0.746 (0.004)		˂0.001^*^ (0.684)	
	F	72.22 (38.24)	79.43 (40.67)	55.30 (26.40)	84.60 (48.61)					

Footnote: Data are presented as the mean (SD), with p-values and partial eta squared (η^2^p) values.

VM: Vastus medialis, VL: Vastus lateralis, RF: Rectus femoris, ST: Semitendinosus, BF: Biceps femoris, MG: Medial gastrocnemius, LG: Lateral gastrocnemius, TA: Tibialis anterior, P: preferred walking speed, F: Fast walking speed.

^*^Indicates statistical significance.

**Fig. 3:**
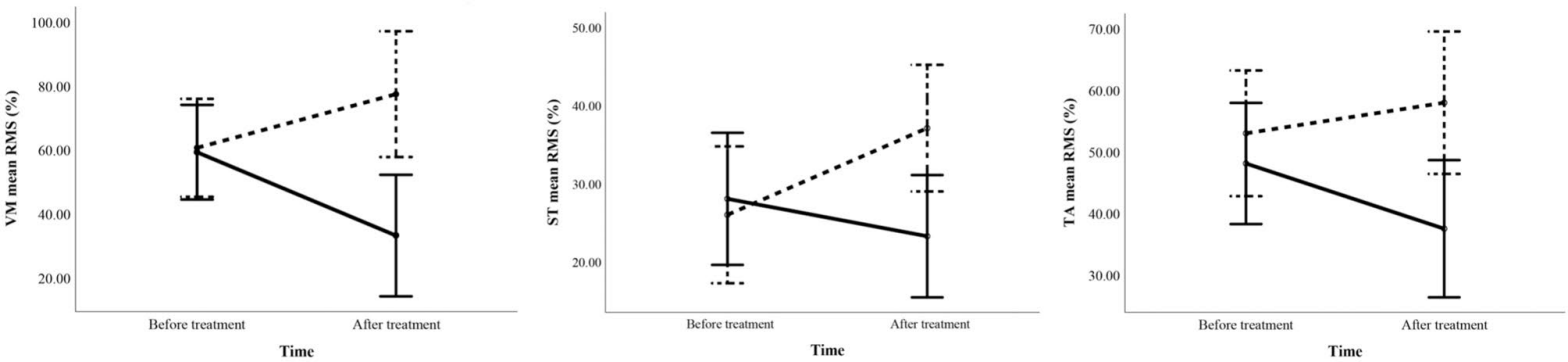
Mean root mean square (RMS) values of the vastus medialis (VM), semitendinosus (ST), and tibialis anterior (TA) in the IASTM group (solid line) and sham group (dashed line) before and after treatment. A significant group*time interaction indicates differing trends between the groups over time. Specifically, IASTM resulted in a reduction in muscle activity, whereas the sham intervention led to an increase.

Mixed ANOVA revealed a significant group effect for VM (p = 46, η^2^p = 0.139), along with significant group*time interactions for VM, ST, and TA muscles (p = 02, η^2^p = 0.309; p = 0.031, η^2^p = 0.161; p 0.036, η^2^p = 0.165, respectively). Given the absence of baseline differences, the VM group effect reflects a treatment-related change. Moreover, the significant interaction terms indicate that the IASTM and sham groups responded differently over time. As shown in Fig. 3, the IASTM group exhibited a post-treatment reduction in mean RMS values, whereas the sham group demonstrated increased muscle activation. No significant main or interaction effects were found for the co-contraction indices across any muscle pairs.

**Fig. 4:**
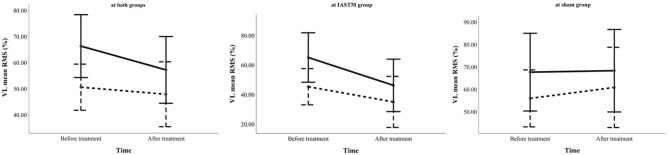
Mean root mean square (RMS) values of the vastus lateralis (VL) when both groups are analyzed together (left graph), in the IASTM group (middle graph), and in the sham group (right graph), across two walking speeds: fast (solid line) and preferred (dashed line) before and after treatment. There was a significant speed*time interaction, indicating that the decline in muscle activity was steeper at the fast-walking speed (left graph). When analyzed separately for each group, the IASTM group exhibited greater reductions in muscle activity at both fast and preferred speeds, whereas the sham group showed a different trend.

With respect to walking speed, a significant main effect of walking speed (p < 0.001) was found for all EMG variables, with both RMS amplitudes and co-contraction indices elevated during fast walking compared to preferred speed. A significant speed* time interaction was also identified for VL (p = 0.0 η^2^p = 0.198), indicating that pre–post changes in VL activation differed depending on walking speed across groups. To illustrate the observed interaction, Fig. 4 includes one combined plot and two group-specific plots, showing pre–post VL activation under both gait conditions. The IASTM group exhibited reduced VL activity following treatment at both speeds, with a more pronounced decline during fast walking. In contrast, the sham group showed increased VL activation in both conditions post-intervention.

### Correlation analysis

Table 4 presents the correlation coefficients. VAS scores showed small to moderate positive associations with VM and RF activation at both preferred and fast walking speeds, as well as with BF activation during fast walking—indicating that higher pain levels were associated with elevated EMG activity in these muscles. In contrast, VAS scores demonstrated moderate negative correlations with knee extensor, knee flexor, plantar flexor, and dorsiflexor strength, suggesting that greater pain intensity was linked to reduced muscle strength. LAI scores showed small negative correlations with knee extensor and flexor strength, reflecting an association between increased functional impairment and lower strength levels.

**Table 4:** Correlation coefficients (r-values) between EMG variables (mean RMS and co-contraction indices), VAS, LAI, and lower limb strength.

**Variables**	**VAS**	**LAI**	**Extension** **strength**	**Flexion strength**	**Plantarflexion strength**	**Dorsiflexion strength**
**VM** (Preferred speed)	0.320^*^	−0.045	−0.239			
**VM** (Fast speed)	0.308^*^	−0.033	−0.257			
**VL** (Preferred speed)	0.156	−0.216	−0.225			
**VL** (Fast speed)	0.144	−0.196	−0.214			
**RF** (Preferred speed)	0.325^*^	0.170	−0.418^**^			
**RF** (Fast speed)	0.286^*^	0.202	−0.405^**^			
**ST** (Preferred speed)	0.126	−0.108		0.029		
**ST** (Fast speed)	0.133	−0.098		−0.030		
**BF** (Preferred speed)	0.242	0.044		−0.192		
**BF** (Fast speed)	0.313^*^	0.051		−0.196		
**MG** (Preferred speed)	0.087	−0.215			−0.279^*^	
**MG** (Fast speed)	0.173	−0.061			−0.276^*^	
**LG** (Preferred speed)	−0.055	−0.180			−0.063	
**LG** (Fast speed)	0.001	−0.090			−0.087	
**TA** (Preferred speed)	0.154	−0.081				−0.244
**TA** (Fast speed)	0.257	−0.077				−0.259
**VM-ST** (Preferred speed)	0.132	−0.090				
**VM-ST** (Fast speed)	0.060	−0.40				
**VL-BF** (Preferred speed)	0.109	−0.155				
**VL-BF** (Fast speed)	0.127	−0.085				
**TA-MG** (Preferred speed)	−0.005	−0.018				
**TA-MG** (Fast speed)	0.048	0.017				
**TA-LG** (Preferred speed)	0.143	−0.018				
**TA-LG** (Fast speed)	0.210	0.024				
**VAS**	-	0.272^*^	−0.442^**^	−0.350^**^	−0.350^**^	−0.300^*^
**LAI**	0.272^*^	-	−0.290^*^	−0.270^*^	−0.237	−0.202

Footnote: VM: Vastus medialis, VL: Vastus lateralis, RF: Rectus femoris, ST: Semitendinosus, BF: Biceps femoris, MG: Medial gastrocnemius, LG: Lateral gastrocnemius, TA: Tibialis anterior. VAS: visual analog scale; LAI: Lequesne algofunctional index.

* indicates p < 0.05, ** indicates p < 0.001.

Most RMS values exhibited negative correlations with corresponding strength measures. Among these, RF activation at both walking speeds showed significant moderate inverse relationships with knee extensor strength, and MG activation demonstrated significant small negative correlations with plantar flexor strength. These findings suggest that higher muscle activation may be associated with diminished strength levels.

## Discussion

This study investigated the effects of a short-term intervention comprising four sessions of IASTM combined with conventional strengthening and stretching exercises, compared to a sham treatment plus exercise, on clinical outcomes and lower limb muscle activation during walking. Given the influence of walking speed on EMG signals, treatment effects were evaluated under both preferred and fast walking conditions to capture neuromuscular responses across varying functional demands. Consistent with our hypotheses, the findings indicated that IASTM combined with exercise significantly improved clinical outcomes—including reductions in pain and functional disability, and gains in muscle strength—while also reducing excessive muscle activation, particularly under the heightened neuromuscular demands of fast gait.

Regarding clinical outcomes, both groups demonstrated significant improvements in pain intensity, strength of the knee flexors and extensors, ankle plantar flexors, and self-reported disability—highlighting the therapeutic benefits of exercise, as even the sham plus exercise group exhibited measurable gains. However, the presence of significant group*time interaction effects indicated that these improvements were more pronounced in the IASTM group, supporting the hypothesis that targeted soft tissue mobilization amplifies symptom relief and functional recovery beyond exercise alone. These findings align with previous research underscoring the efficacy of IASTM in reducing pain, increasing muscle strength and joint range of motion, and improving overall health status^24,26,27,41^. Proposed physiological mechanisms include increased local blood flow, reduced tissue viscosity, disruption of fascial adhesions, realignment of collagen fibers, and facilitation of the inflammatory resolution process^20–23^. In the present study, the application of IASTM over pre-articular structures—including the quadriceps tendon, patellar margins, and surrounding musculature—appeared to enhance tissue responsiveness and contribute to functional recovery.

From a neuromuscular perspective, a notable finding was the reduction in muscle activation following IASTM treatment. Significant group-by-time interactions (Fig. 3) revealed decreased EMG activity in the VM, ST, and TA muscles within the IASTM group, whereas the sham group exhibited increased activation in these same muscles. This finding is clinically meaningful, as individuals with KOA often exhibit heightened muscle activation as a compensatory response to pain-induced neuromuscular inhibition and compromised joint stability. Although such compensatory mechanisms may enhance short-term joint support, they concurrently elevate intra-articular pressure and may accelerate degenerative changes over time^9–17^. In this context, the observed reduction in muscle activity post-IASTM likely reflects a modulation of neuromuscular output toward more efficient muscle function—potentially reducing joint loading and contributing to long-term joint preservation. These findings align with the limited body of literature examining the neuromodulatory effects of soft tissue release techniques—including manual therapy, massage, and foam rolling—which have demonstrated reductions in muscle activity and α-motoneuron excitability^45–48^. In contrast, the increased EMG activation observed in the sham group suggests that exercise alone, in the absence of myofascial intervention, may perpetuate neuromuscular overactivity and exacerbate intra-articular stress. Importantly, the reductions in EMG activity observed in the IASTM group occurred alongside concurrent improvements in strength and pain levels. This constellation of changes, supported by correlation analyses, suggests that lower EMG amplitudes did not signal neuromuscular inhibition, but rather indicated a shift toward more efficient neuromuscular response—characterized by enhanced muscle strength and reduced reliance on compensatory activation to maintain joint integrity during gait. Nevertheless, it is important to acknowledge that while surface EMG provides valuable insight into muscle activation, it does not directly assess motor unit recruitment or central neural drive. Therefore, interpretations regarding central mechanisms remain speculative and should be approached with caution.

Another aspect of this study was the exploratory correlation analysis, which provided additional insight into the relationships among pain, muscle strength, and EMG activity. Pain scores showed positive correlations with EMG activity—particularly in the VM and RF muscles at both walking speeds, and in the BF muscle during fast walking—suggesting that higher perceived pain may be associated with increased muscle activation. In contrast, pain scores were negatively correlated with strength measures across all major muscle groups, and similar inverse relationships were observed between strength and EMG activity. These findings suggest that individuals experiencing higher pain levels tend to exhibit reduced muscle strength, potentially due to pain-induced neuromuscular inhibition, which impairs force generation^6,7^. As a compensatory response, patients may increase muscle recruitment to offset strength deficits—a pattern most pronounced in the knee extensors (VM, RF), which act as primary stabilizers, particularly at higher walking speeds where joint control demands are greater. Post-treatment improvements in strength, alongside reductions in pain and excessive muscle activation, point to a shift toward more efficient neuromuscular coordination. In the IASTM group, as participants experienced pain relief and strength recovery, their reliance on excessive activation diminished, leading to enhanced movement efficiency. However, the absence of significant correlations involving co-contraction indices suggests that additional interventions or prolonged treatment may be required to elicit measurable adaptations in intermuscular coordination strategies.

Beyond neuromuscular and strength-related outcomes, the intervention also appeared to influence functional status. A positive correlation between pain and LAI scores indicated that higher pain levels were associated with greater self-reported disability, whereas pain reductions corresponded with improved LAI scores post-intervention. Additionally, LAI scores were negatively correlated with knee extensor and flexor strength, consistent with the scale’s focus on knee-specific functional capacity. These relationships underscore the functional relevance of knee muscle strength in determining LAI outcomes and emphasize its role as a key determinant of functional status and perceived disability in individuals with KOA.

In this study, treatment effects were also assessed under fast walking conditions. Although individuals with KOA typically adopt slower gait patterns in daily life, situations requiring rapid movement—such as navigating environmental obstacles or responding to urgent events—can exacerbate pain and increase injury risk. As expected, EMG activity, including mean RMS values and co-contraction indices, was significantly elevated during fast walking compared to preferred speed. This finding aligns with previous research indicating that higher gait speeds impose greater biomechanical demands, necessitating increased neuromuscular control and joint stabilization^28^. However, such elevated activation may intensify joint stress, underscoring the importance of interventions that enhance muscle efficiency while maintaining joint integrity in individuals with KOA. Notably, a significant speed-by-time interaction was observed for the VL muscle, indicating that pre–post changes in activation were influenced by gait speed across groups. To better visualize group-specific trends, results were presented separately for each group in Fig. 4. VL activation decreased in the IASTM group at both walking speeds, with a more pronounced reduction during fast gait. In contrast, the sham group exhibited increased VL activity post-intervention. These patterns underscore the potential of IASTM to modulate excessive muscle activation, particularly under the heightened neuromuscular demands of fast walking.

While this study offers valuable insights into the effects of IASTM combined with exercise therapy on muscle activation in individuals with moderate KOA, some limitations should be acknowledged. Although the number of treatment sessions was based on prior studies demonstrating the immediate and short-term effects of IASTM^26,40,41^, and supported by the significant interaction effects observed in this study, additional sessions may still be required to elicit more robust neuromuscular adaptations. Moreover, as outcomes were assessed 48 hours post-intervention, longer-term follow-up would be necessary to evaluate the durability of the observed changes.

Another consideration is the inclusion of participants with both tibiofemoral and patellofemoral osteoarthritis, identified through the Kellgren–Lawrence grading system and Clarke’s test. This combined presentation, commonly observed in clinical settings, was addressed by implementing the IASTM protocol designed to target both peri-articular tissues and key knee musculature. However, future studies may benefit from stratifying patients by OA subtype to better clarify intervention-specific effects.

Additionally, EMG data in the present study were analyzed across the entire stance phase of walking, which represents the load-bearing period of gait and holds clinical relevance in individuals with KOA. While this approach captures meaningful muscle activation levels, subdividing the stance phase into distinct segments or applying time-normalized analyses may yield more detailed insights and should be considered in future research. Moreover, assessing neuromuscular responses across a broader range of functional tasks—such as stepping or sit-to-stand movements—could offer a more comprehensive understanding of treatment efficacy in everyday activities.

Finally, although the correlation analyses provided exploratory insight into relationships among variables, the findings should be interpreted with caution, as Pearson’s method assesses the strength and direction of associations but does not imply causality. Future studies with larger sample sizes may benefit from applying multiple regression to better account for potential confounding factors and to clarify the relative contribution of each variable to clinical outcomes.

## Conclusions

This study demonstrated that both intervention groups—IASTM combined with exercise and sham IASTM combined with exercise—led to improvements in pain, strength, and disability among patients with KOA, underscoring the clinical value of therapeutic exercise, as meaningful benefits were also observed in the sham condition. However, the IASTM group showed more pronounced improvements across these outcomes, accompanied by significant reductions in muscle activity.

Although elevated muscle activation is commonly observed in KOA as a compensatory response to pain-induced inhibition and joint instability, its concurrent reduction— alongside improvements in strength, pain, and disability—suggests that IASTM may promote more efficient neuromuscular function aligned with clinical recovery. This improvement may contribute to reduced joint loading and potentially slower disease progression—a hypothesis that warrants further longitudinal investigation.

Moreover, while fast walking typically elicits elevated EMG activity due to greater biomechanical and stability demands, the IASTM group demonstrated a post-treatment trend toward reduced vastus lateralis activation compared to the sham group, suggesting improved movement efficiency even under high-demand gait conditions. To better elucidate these findings, future studies could consider additional treatment sessions. Overall, the results support the incorporation of soft tissue mobilization techniques such as IASTM into multimodal rehabilitation strategies for KOA, with potential benefits for both neuromuscular control and symptom management.

## Data Availability

The datasets used and/or analyzed during the current study are available from the corresponding author upon reasonable request.

## Abbreviations

KOA: Knee osteoarthritis
EMG: Electromyography
IASTM: Instrument-assisted soft tissue mobilization
RMS: Root mean square
LAI: Lequesne algofunctional index
VAS: Visual analog scale
BMI: Body mass index
MVIC: Maximum voluntary isometric contraction
RF: Rectus femoris
VM: Vastus medialis
VL: Vastus lateralis
ST: Semitendinosus
BF: Biceps femoris
TA: Tibialis anterior
MG: Medial gastrocnemius
LG: Lateral gastrocnemius
